# Supplementary figures and images for: Structure and neutralization mechanism of a human antibody targeting a complex Epitope on Zika virus
Source: PLoS Pathog. 2023 Jan 10;19(1):e1010814. doi: 10.1371/journal.ppat.1010814 (PMC9870165; doi:10.1371/journal.ppat.1010814)

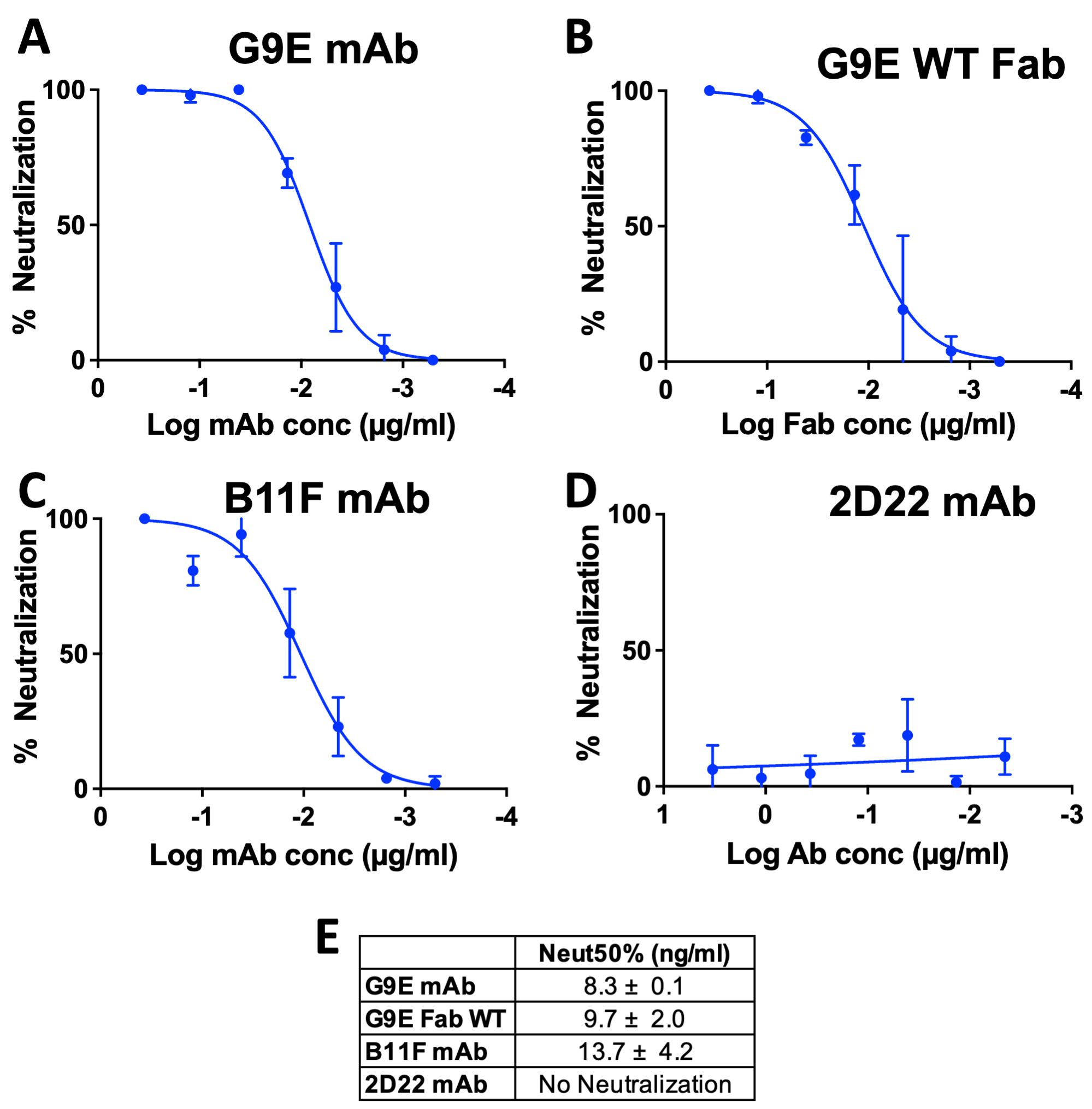

Supplement: S1 Fig — Representative ZIKV neutralization curves for G9E mAb (A) and G9E Fab (B), along with previously characterized ZIKV specific (C, B11F) and DENV2 specific (D, 2D22) mAbs, are shown. G9E mAb and G9E Fab were expressed in Expi293 mammalian cells and purified from the cell culture medium by MabSelect resin or nickel-nitrilotriacetic acid agarose resin, respectively. G9E Fab retains neutralization activity against ZIKV, similar to the parent G9E mAb. Error bars for each data point are standard deviations of the technical replicates. (E) EC50 values obtained from nonlinear sigmoidal 4PL curve fitting are shown. (TIF) [file ppat.1010814.s001.tif]

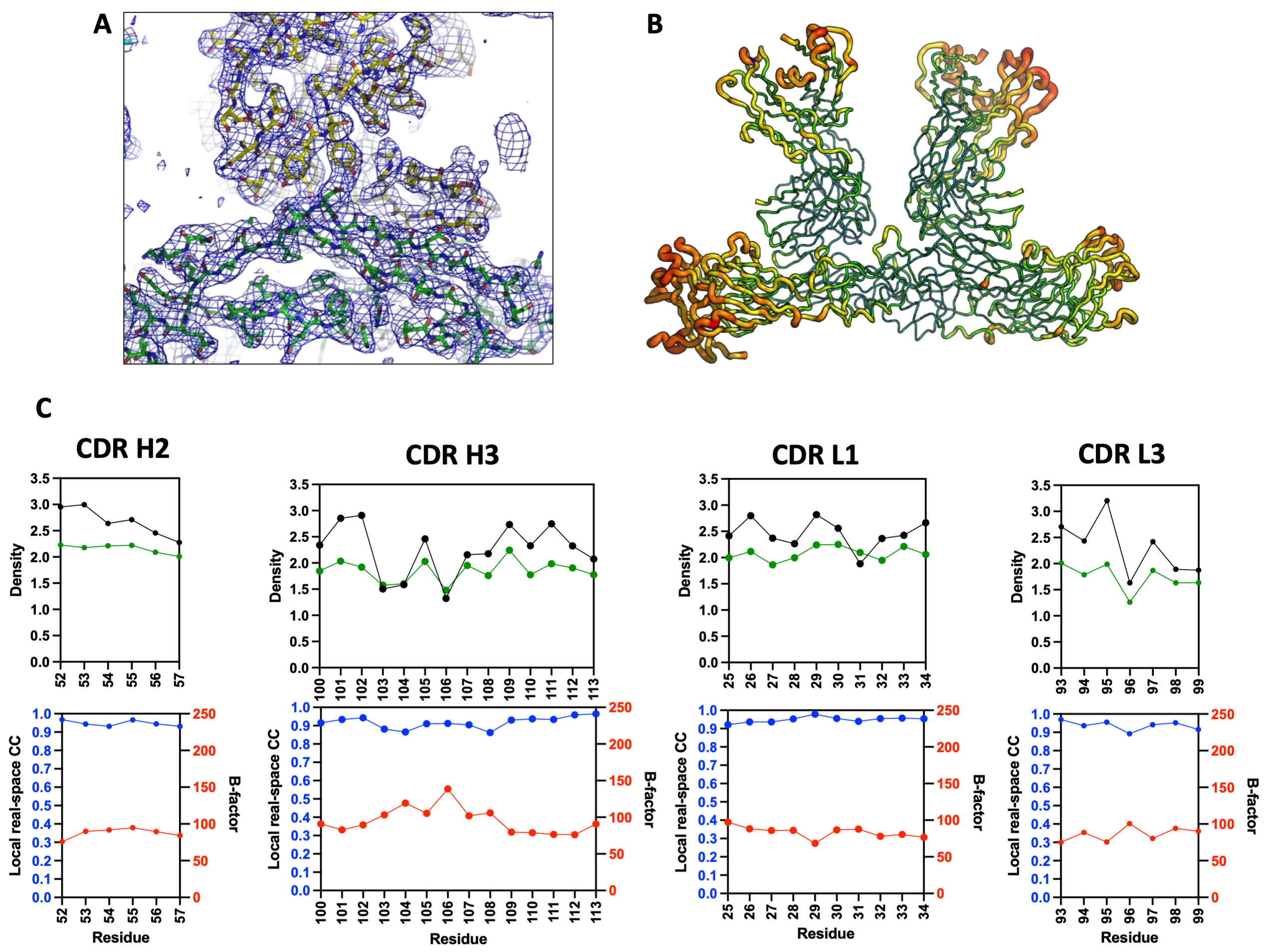

Supplement: S2 Fig — (A) Representative electron density map of G9E Fab/ZIKV E complex. An initial 2Fo-Fc electron density map (contour 1.0 sigma) of the ZIKV E/G9E complex illustrates that the starting phases obtained by molecular replacement were of excellent quality to reveal the nature of the interaction between G9E and ZIKV-E-protein. ZIKV E-protein (green) G9E Fab (yellow) are shown as sticks. (B) Thermal parameter distribution in ZIKV E/G9E Fab shown as B-factor “putty”. The isotropic B-factors are depicted on the structure as spectrum range from 28.9 Å2 (blue, lowest B-factor) to 238.7 Å2 (red, highest B-factor), with the ribbon radius increasing from low to high B-factor. The mean B-factor was 120.67 Å2. The lowest B-value was observed in the interfacing region between DII of E protein and the CDR regions of G9E Fab, where the electron density is well resolved. (C) Molprobity multicriterion-plot for CDRs. The likelihood-weighted 2mFo-DFc map and the Fc map calculated from the model were compared and real-space correlation coefficient for each residue were obtained. Comparison of the 2mFo-DFc map, the Fc map, the real-space CC and the B-factor for each of reside in CDR loops are shown. (TIF) [file ppat.1010814.s002.tif]

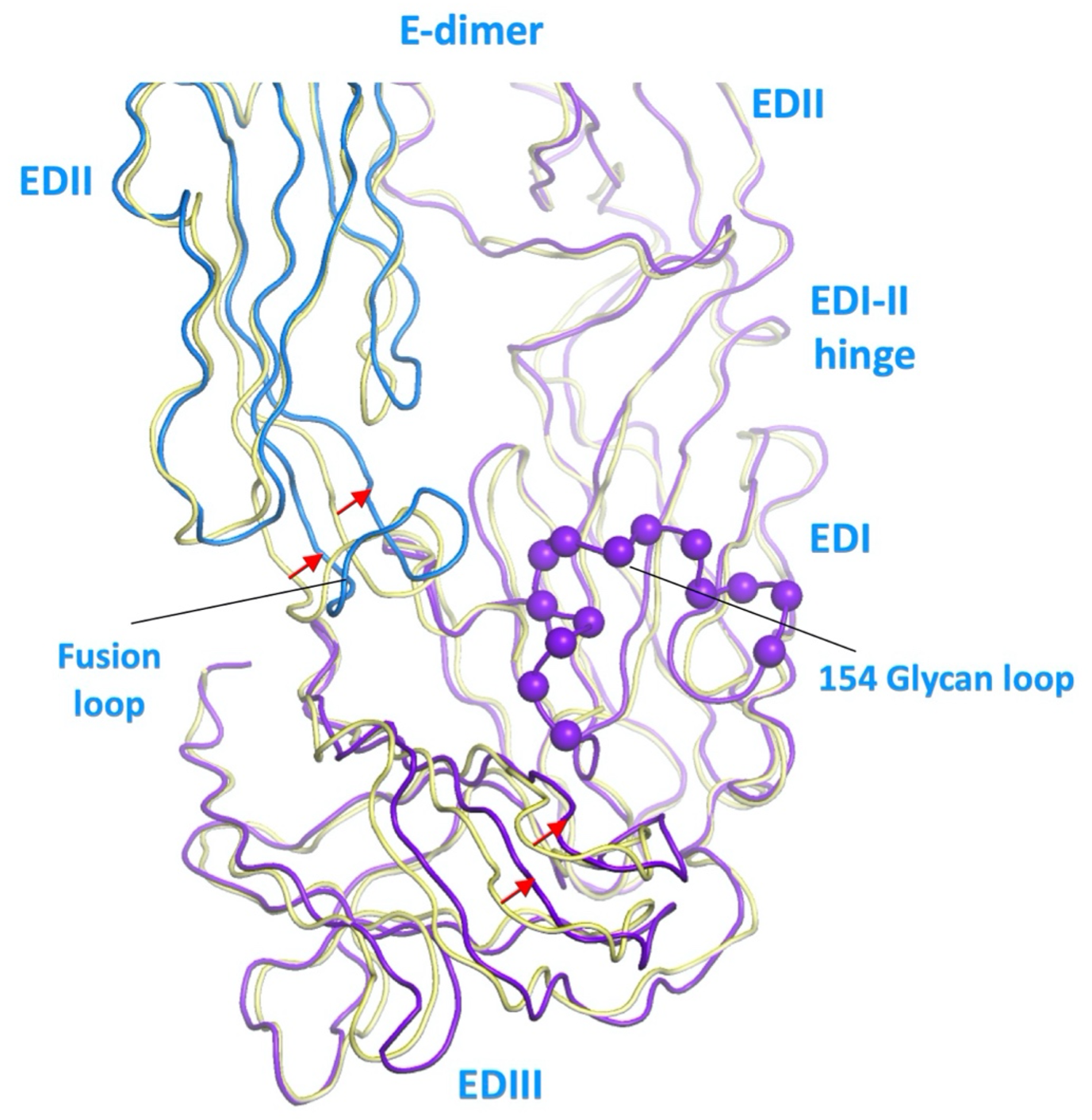

Supplement: S3 Fig — Structural superposition of the E-protein conformation of the template structure used for molecular replacement (PDB ID: 5JHM, yellow) and the E-protein conformation observed in complex with G9E (protomer 1—blue; protomer 2—purple). G9E induces a 2 Å inward movement of the fusion loop (blue strand, notated by red arrows) towards the EDI glycan loop (purple spheres) of the neighboring E-protein. G9E also causes a 3 Å inward movement of EDIII (purple strand, notated by the red arrow) towards its EDI glycan loop (purple spheres). These movements cause an increase in the E-dimer interface. (TIF) [file ppat.1010814.s003.tif]

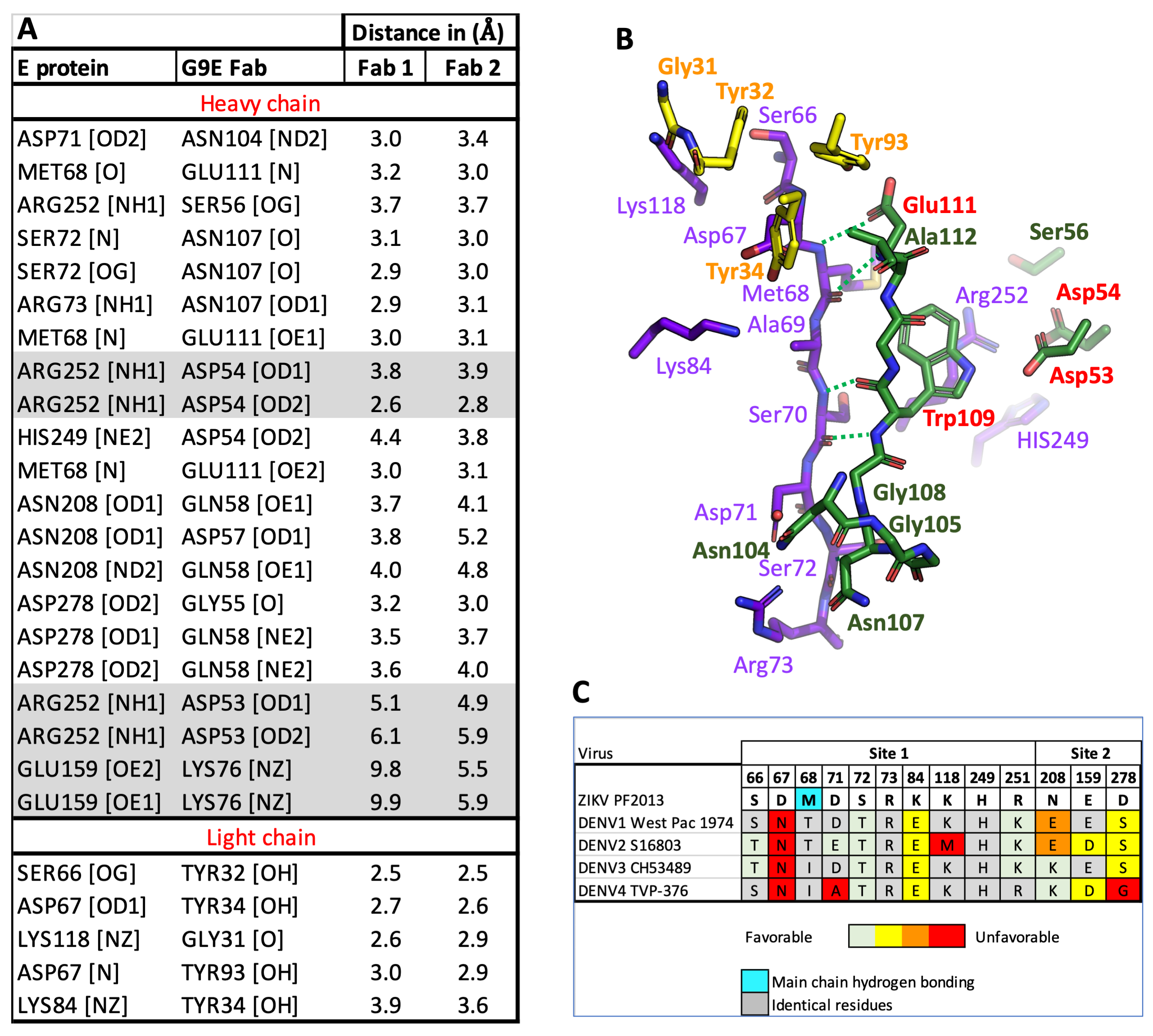

Supplement: S4 Fig — A. Summary of interaction distance between E and G9E residues in the crystal structure of ZIKV E/G9E complex. A donor-acceptor atom distance of 4 Å between E and one of the Fab was considered as a H-bond. A distance of 6 Å was considered for a salt bridge. B. Close-up view of the interacting residues in E protein (purple) and G9E heavy (green) and light chains (yellow). The color of the residue name and number is matched to the carbon skeleton of amino acid. The main chain hydrogen bonding interaction between E and G9E are shown as dotted green lines. The heavy chain paratope residues selected for site-directed mutations are highlighted in red text. C. Amino acid conservation analysis within the G9E binding site. G9E interacting residues in ZIKV E protein was compared to the four serotypes of the DENV E proteins. ZIKV E protein residue number and name in single letter code are provided on the top rows. Based on the amino acid properties, DENV residues are categorized from favorable (green) to unfavorable (red). Identical residues are colored in grey. ZIKV E residue involved in main chain hydrogen bonding interaction is shown in cyan. DENV sequences were retrieved from NCBI using the accession codes provided within the parentheses: DENV1 (P17763), DENV 2 (GU289914), DENV3 (AAB69126), and DENV4 (AGS14893). The N-linked glycosylation at position 67 was predicted to sterically block G9E binding. (TIF) [file ppat.1010814.s004.tif]

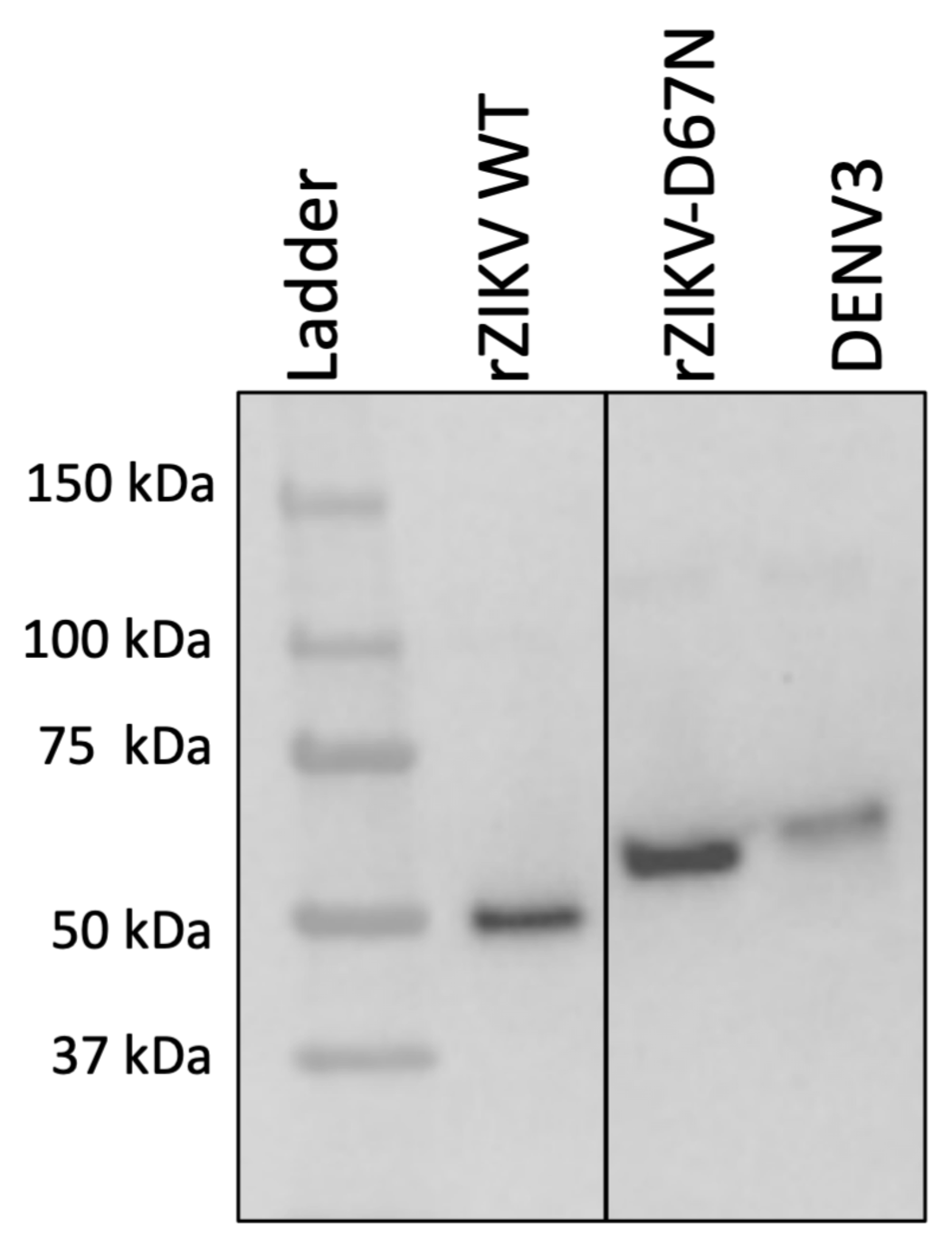

Supplement: S5 Fig — WT rZIKV retains a glycosylation site at position 154 on the envelope protein while DENV3 has two glycosylation sites at positions 67 and 154. rZIKV-D67N was created by introducing a glycosylation motif (DMA to NTT) at position 67–69. To indirectly assess glycosylation status, E-proteins were immunoprecipitated from Vero cells infected with WT rZIKV, rZIKV-D67N, or DENV3 and detected by western blot using flavivirus mAb 4G2 as primary antibody followed by HRP-conjugated goat anti-mouse IgG as secondary antibody. rZIKV-D67N exhibited a higher molecular weight E protein compared to WT rZIKV, consistent with the presence of an additional N-linked glycan. (TIF) [file ppat.1010814.s005.tif]

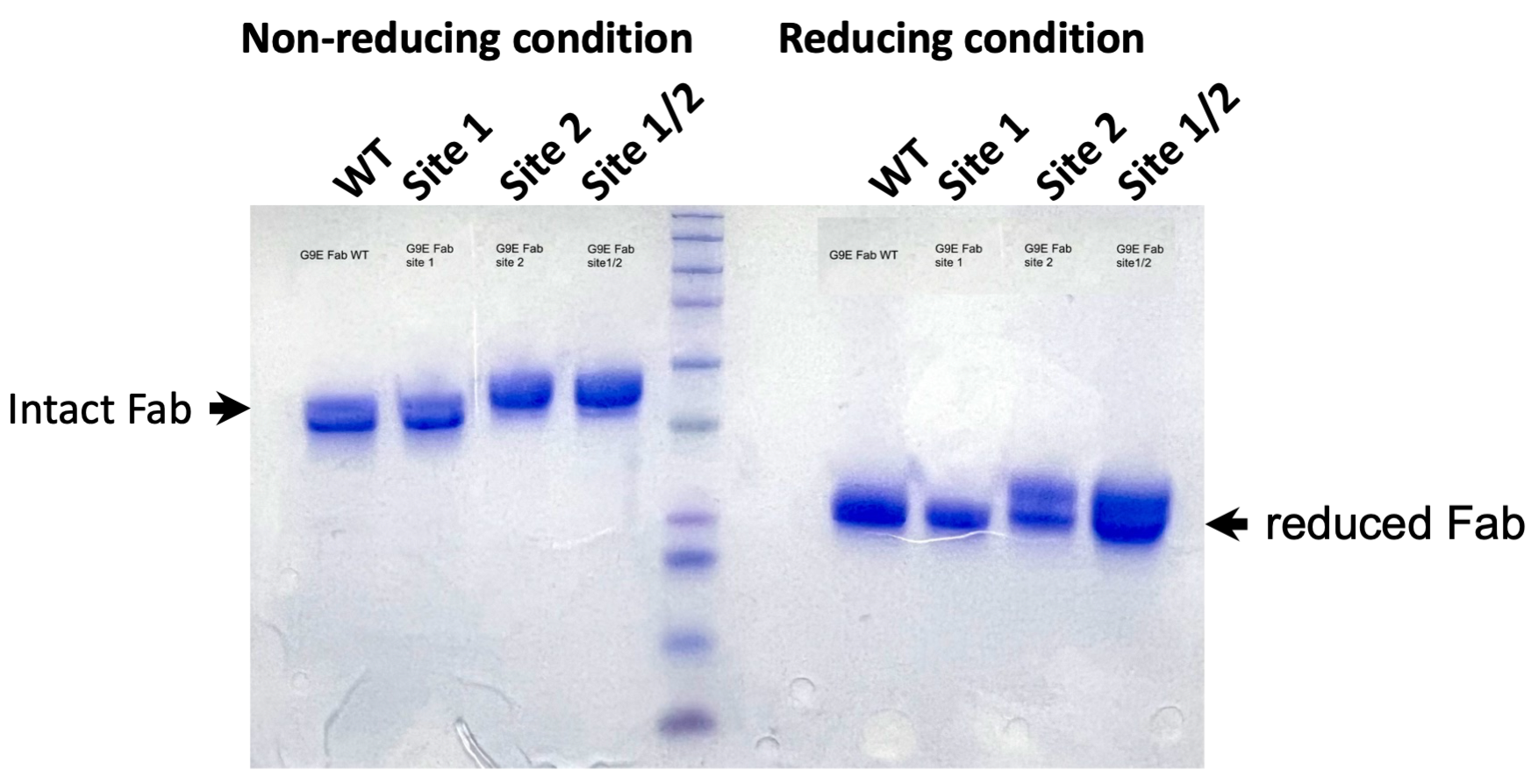

Supplement: S6 Fig — G9E WT and paratope mutant Fabs were expressed in Expi293 mammalian cells and purified by Ni-NTA resin. Coomassie-stained SDS-PAGE run under reduced and non-reduced condition show the band corresponding to intact Fab and reduced Fab fragments. (TIF) [file ppat.1010814.s006.tif]
