# Supplementary material for: Structure and neutralization mechanism of a human antibody targeting a complex Epitope on Zika virus
Source: PLoS Pathog. 2023 Jan 10;19(1):e1010814. doi: 10.1371/journal.ppat.1010814 (PMC9870165; doi:10.1371/journal.ppat.1010814)
Supplement: Attachment — Submitted filename: Response to reviews.doc [file ppat.1010814.s010.doc]

**Response to reviewers’ comments [PPATHOGENS-D-21-00433]**

All reviewer comments are in regular text and our responses are in *italics*.

**Reviewer 1**

1) By introducing a glycosylation site at D67 of ZIKV, the authors show loss of G9E mAb binding. They have compared this with other human mAbs – Z20, ZKA190, ZIKV117, A9E and C8, where they suggest that the mutation did not affect the binding of either ZKA190, A9E, C8 and surprisingly ZIKV117. ZIKV117 is a dimer cross-linking mAb and it has been shown by shot-gun mutagenesis experiments that the residue D67 is critical for ZIKV117 binding. I would expect that the introduction of a glycosylation site (D67N) would certainly hinder ZIKV117 binding. Upon thorough analysis, I realized that the authors have used the PDB Ab sequence of 5UHY to synthesize the gene and express/purify it for their experiments. It has been clearly stated in the paper from Hasan et al., that the mAb fitted in the cryo-EM map was derived from ZV67, a DIII binding Ab. I would suggest to remove the studies on ZIKV117 or perform the experiments with another DII cross-linking mAb, where the sequence is available.

*Response: We thank the reviewer for pointing out that the sequence deposited in the PDB entry (5UHY by Hasan et al.) for the ZIKV117 structure is actually for ZV-67, a ZIKV EDIII specific mouse mAb, and the entry sequence is not related to ZIKV117. Accordingly, we have changed all references to ZIKV117 to indicate that we have used a humanized version of EDIII-specific ZV-67 mAb in our study.*

*In lines 336-338: we have added the following texts to clarify that a humanized anti-EDIII mAb ZV67 was used in our research. “A humanized version of ZIKV specific mouse EDIII mAb (HuZV-67) in IgG1 format was generated using the sequence obtained from PDB entry 5KVG.”*

*In lines 150-153: we have changed the text to read “As predicted, G9E and Z20 was unable to bind or neutralize rZIKV-D67N (Fig. 3B and C). Human mAbs that bind to EDIII (ZKA190 and Hu-ZV67), EDI (A9E), and the E dimer dependent epitope conserved between DENVs and ZIKV (EDE C8) bound and neutralized both WT and rZIKV-D67N (Fig. 3B and C).”*

*In Figure 3B and 3C, we changed the name “huZIKV-117” to huZV-67”*

2) L54-L56: The authors might consider to discuss about why some E protein specific Abs potently neutralize and others don’t in detail and how they answered this question with their experiments.

*Response: We have revised the introduction to expand on this point and the contribution of the current study to our understanding of ZIKV-antibody interactions (see lines 53-71).*

3) L77-80: Was NCS used during refinement? Two copies similar because of NCS?

*Response: During the structure refinement, we used torsional angle noncrystallographic symmetry (NCS) restraints rather than global-local atomic distances to allow local differences between the NCS-related molecules. The high similarity between the two copies of the NCS related molecules suggests that our crystal structure captures the most preferred mode of G9E binding to ZIKV E protein. We have included a text in the methods section to clarify that Group-B factors and torsional angle (NCS) restraints were used throughout the refinement cycles (see line 379-380).*

4) L80-83: Comparison with the E-dimer on the virus will be useful here rather than comparing with another crystal structure (5JHM). Can G9E bind to the E dimer conformation on the mature virus? Are there interactions with the fusion loop?

*Response:  Crystal structures of soluble E dimer and CryoEM structures of E dimer on the virus are structurally similar, but some conformational differences between these structures have been described previously [PMID: 15341726]. The soluble E protein typically adopts a flat conformation that differs from the conformation of the E protein structures on the mature virion. The difference is usually* located around *the ED-I/II hinge angle, which supports the E protein's curvature on the virion. In the revised manuscript, we added the cryo-EM structure of ZIKV in complex G9E Fab. Comparisons of the E dimer in the G9E Fab complex determined by the crystal structure and the cryo-EM structure showed that G9E binding modes are highly similar. Yet, there was a global domain shift in EDII in line with the curved conformation observed on the mature virion (see lines 107-113 and Fig. 2E).* *As shown in Figure 5 and Supplementary Figure 4, G9E does not interact with fusion loop residues.*

5) Figure 1: It would help the readers if the three E protein domains are colored different. In 1C, color the symmetry equivalent sites of 117 and 195 epitopes. The purpose of 1D?

*Response: We agree with the reviewer's suggestions and revised figures 1C and 1D with the E domains shown in different colors. The revised figures also show the symmetry equivalent sites for ZIKV-117 and ZIKV-195. The purpose of figure 1D is to map an immunodominant location on ZIKV E-raft by combining the EDII targeting antibody epitopes. Figure 1C and Figure 3D demonstrate that an antigenic region defined by EDII and EDI-glycan loop and EDI/II hinge region is a significant target of serum neutralizing antibodies among Zika patients.*

6) Glycan blocks binding of G9E and other ZIKV potent antibodies: It would be helpful to compare the epitope using a multiple sequence alignment. What happens with G9E binding when D67 is mutated to alanine? What are the critical epitope residues on ZIKV for G9E binding? What is the G9E epitope conservation between ZIKV and DENV? I would expect that G9E might not be able to neutralize DENV upon de-glycosylation of DENV at position 67.

*Response: We thank the reviewer for the thoughtful suggestion. The figure below shows that the G9E epitope residues between ZIKV and DENV are moderately conserved at site 1 and less conserved at site 2. In the revised manuscript, we have included the amino acid conservation analysis of the G9E epitope in the supplementary Figure 4C.*

*We have not generated a ZIKV E mutant with alanine at position 67. In our ZIKV E/G9E crystal structure, OD1 of D67 (E) form hydrogen bonding interaction with OH of Y34 (G9E:light chain). Our structure predicts that changing alanine for aspartate at position 67 may reduce G9E binding to ZIKV.*

*We agree with the reviewer that it would be interesting to test if G9E can neutralize de-glycosylated DENV at position 67. However, this would require recombinant mutant dengue virus with a mutation at position at 67. As N-linked glycosylation at position 67 is critical for dengue virus infection of cells (Bryant JE et al. PMID: 17543367), this is a difficult mutant to generate for studies.*

7) Please check the comment about ZIKV117 under summary. I have not checked other sequences but would strongly suggest the authors to revise the IgG mAb sequences used for their experiments.

*Response: Please see our response to comment #1.*

8) Figure 4: E259 or G259? Please check the sequence. The labeling of glycan loop might not be correct? Can the site 1 and site 2 mutants be highlighted in figure 4 or in a supplementary figure?

*Response: We thank the reviewer for pointing out this typographical error. E159 is incorrectly labeled as E259. We have fixed this error in the revised figure 5A. As suggested, we have highlighted the G9E paratope mutants we generated to perturb the interaction with site 1 and site 2 epitope residues in Figure 5B and supplementary Figure 4B.*

9) L241; The antigenic site is not exclusively defined by G9E, but by other mAbs like Z20, ZIKV190 and ZIKV117. Can it be discussed why, apart from glycosylation? Is this the least conserved region or more surface accessible?

*Response: The immunodominant antigenic site defined by the above EDII targeting ZIKV mAbs is moderately conserved across flaviviruses and is highly surface accessible (Supplementary Figure 4C). However, the presence of the dengue virus-specific N-linked glycosylation at position 67 masks the epitope, which is likely to reduce the immunogenicity of the site in DENVs. We predict that the corresponding site in other flaviviruses without a glycan at N67 (Japanese encephalitis, West Nile, and Spondweni viruses) is likely to be target of strongly neutralizing and protective antibodies. We have revised the discussion to address this topic (See lines 309–313).*

10) Is it possible to prepare a table of Ab-ZIKV interacting residues and H-bond distances?

*Response: We have added a supplementary table detailing the interacting residues and distances.*

**Minor issues**

11) The Title: “High-resolution structure” in the title is misleading. A high-resolution crystal structure would be around 1.5 A. Might consider changing it to Structure of or Crystal structure and …

*Response: As suggested, we have eliminated “High-resolution” from the title in the revised manuscript.*

12). Abstract: L22. Spanned two E molecules on the viral surface is also misleading as this is a crystal structure of E ectodomain in complex with the Ab.

*Response: In the revised manuscript, we have changed the abstract as we are defining the footprint of G9E with ZIKV by both X-ray crystallography and cryo-EM.*

13) Introduction: L43: remove “ticks” as it sounds like ZIKV is transmitted by ticks

L43: positive-sense SS RNA – may be modify this sentence and include prM/M.

L47: Mature or immature virus?

*Response: We have revised the introduction paragraph to eliminate these ambiguities.*

14) L221: What is MBC?

*Response: MBC refers to memory B-cells. We have clarified the abbreviation MBC in the revised manuscript (line 284).*

15) L297-301: What are the Z-scores of MR solution? What is the R-factor? Was something similar to prime-and-switch phasing performed to minimize the model bias? Was the electron density of the CDR loop back bone and side chains clearly visible? What kind of B-factor refinement was performed? Any TLS refinement?

*Response: As described in the Methods section, we obtained the correct MR solutions by searching for two copies of the four template structures (ensemble 1: ZIKV EDI-EDII (1-301 aa, PDB ID: 5JHM), ensemble2: Fab heavy chain (PDB ID: 4NKI), ensemble3: Fab light chain (PDB ID: 4NKI) and ensemble4: ZIKV EDIII (302-406 aa, PDB ID: 5JHM) using Phaser v 2.7.17. The translation function Z-scores (TFZ) were >8 for 7 of 8 solutions. The log-likelihood gain increased from 116 (1st solution) to 1444 (8th solution) as each component of the solution was added. The details of the EULER angles and TFZs for each MR solution are listed below. We have also listed the final MR solution obtained using the fully assembled ZIKV E/Fab complex as a template.*

**Summary of the MR solutions**

#1 RecE (EDI-EDII) copy 1

SOLU 6DIM ENSE ensemble1 EULER 306.215	10.999	45.473 FRAC 0.03501 -0.01162 0.45807 BFAC -9.31029 #TFZ==9.3

#2 RecE (EDI-EDII) copy2

SOLU 6DIM ENSE ensemble1 EULER 12.513 128.071 178.788 FRAC -0.05628 -0.37260 1.22951 BFAC 10.64873 #TFZ==12.3

#3 G9E-Fab-heavy copy 1

SOLU 6DIM ENSE ensemble2 EULER 353.165 136.327 176.042 FRAC -1.02083 -0.46441 0.51835 BFAC -1.84891 #TFZ==14.7

#4 G9E-Fab-heavy copy 2

SOLU 6DIM ENSE ensemble2 EULER	94.754	0.621 264.885 FRAC -1.00019 0.06003 0.49447 BFAC 0.69703 #TFZ==14.3

#5 G9E-Fab-light copy 1

SOLU 6DIM ENSE ensemble3 EULER 307.337	2.365	54.132 FRAC -0.99714 0.05874 0.50423 BFAC 2.28629 #TFZ==13.0

#6 G9E-Fab-light copy 2

SOLU 6DIM ENSE ensemble3 EULER 2.310 133.453 183.601 FRAC -0.98940 -0.45525 0.50347 BFAC -2.62920 #TFZ==15.5

#7 RecE (EDIII) copy 1

SOLU 6DIM ENSE ensemble4 EULER 272.215	8.231	79.061 FRAC -0.97067 -0.01651 0.51820 BFAC -14.55050 #TFZ==12.4

#7 RecE (EDIII) copy 2

SOLU 6DIM ENSE ensemble4 EULER 23.114 107.261 219.906 FRAC -1.02970 -0.58986 0.29246 BFAC 15.12944 #TFZ==6.8

#1 RecE (RecE EDI-EDII/RecE EDIII/G9E-Fab-heavy/G9E-Fab-light)

SOLU 6DIM ENSE ensemble1 EULER 359.987	0.000	0.000 FRAC 0.00079 0.00000 0.00106 BFAC 8.91604 #TFZ==22.2

*We performed the structure refinements in Phenix. The initial R-factor for the MR solution was 44%. After 3 cycles with a combination of rigid body, XYZ, and group-B refinement strategy, the starting R-factor and R-free were 29.9% and 32.3%, respectively. The final R-factor and R-free reported in the Supplementary table 2 are 23.2 and 25.9%, respectively. Our early refinement strategy included torsion angle simulated annealing to eliminate model bias. Electron density maps for the regions, for example, the E 154 glycan loop and the CDR loops, which were absent in the template model, became interpretable. We used Group-B factors and torsional angle noncrystallographic symmetry (NCS) restraints* *throughout the refinement cycles, and the final refinement cycles had TLS refinement utilizing a total of 6 TLS groups (1 TLS group per chain). The temperature factors for both heavy and light chain CDR loops are low, and the electron density corresponding to these regions are interpretable to confidently assign main and side-chain atoms. A representative electron density map shown in supplementary figure 2 provides the snap-shot of the interface between the G9E Fab heavy chain and EDII of E protein. We now added two new images in supplementary figure 2 to show real-space correlation and thermal parameter distribution in ZIKV E/G9E Fab complex. As shown in Supplementary 2B, quality of the density and the model fitting the density in the CDR loop regions are high. The above details are now added to the methods section (lines 362–387).*

16) Supplementary Figure 2: phases by MR are of excellent quality? What are the R-factors?

*Response: As described above, the starting MR phases were of high quality and allowed rebuilding of the correctly placed model and build outside template model, including the G9E CDRs and the E154 glycan loop after initial refinement cycles. Consistent with high B-factors, we could not trace the N and C-terminal residues due to the considerable flexibility in these regions (Supplementary Figure 2B). This includes C-terminal linker and His-tag residues (10 aa) in E protein, C-terminal heavy chain residues (10 aa), and light chain N-terminal (1 aa) and C-terminal (3 aa) residues.*

*As described above, the starting R-factor and R-free were 29.9% and 32.3%, respectively. The final R-factor and R-free reported in the Supplementary Table 2 are 23.2 and 25.9%, respectively.*

17) Supplementary Figure 4B: G9E mAb binding on the raft and not on whole virus?

*Response: This figure has been eliminated in the revised manuscript as we now describe the cryo-EM structure of ZIKV in a complex with G9E Fabs. As shown in Figure 2B, the cryo-EM map revealed that there are, in total, 180 Fabs binding to one Zika virus particle.*

18) Supplementary Table 2: What is the R-merge at 3sigma cutoff? Perhaps the resolution is over-estimated? How were the R-free reflections selected? (Thin shells is preferred because of the presence of NCS).

*Response: A summary of reflection intensities and R-factors by shells are listed below, with the row corresponding to R-merge at 3-sigma (0.997) highlighted in yellow. Historically, R-merge was used to estimate the non-isomorphism of data collected on film using several different crystals. However, in our experiment, we collected the complete data set from a single frozen crystal with a high overall redundancy of 7.5. Moreover, Rmerge is a poor predictor of precision of X-ray diffraction data as it tends to be higher with higher data redundancy (J. Appl. Cryst. 30, 203-205 and in J. Appl. Cryst. 34, 130-135). In our diffraction experiment, collection of data with high redundancy improved the averaged data quality as suggested by the low Rpim (redundancy independent precision-indicating merging R factor) and high CC½ (higher correlation coefficient between random half-datasets) and high CC* (correlation estimate of the averaged intensities) by each shell. To determine the data quality and resolution cut-off, we used the correlation coefficient between random half-datasets CC½ (>0.2), mean signal/noise (1.0), and completeness (>80%) in the high-resolution shell as a guideline. As presented in the data collection statistics, CC½ (0.62), mean I/sigma (1.4), and completeness (94.7%) exceed these conventions in the high-resolution shell. For R-free, we used 5% of randomly selected reflections assigned to the Free-R set by Uniquify (implemented within the AIMLESS pipeline in CCP4).*

```
Shell Lower Upper Average      Average     Norm. Linear Square
 limit    Angstrom       I   error   stat. Chi**2  R-fac  R-fac  Rmeas   Rpim  CC1/2    CC*
      50.00   9.21  1464.6    20.9    10.9  2.467  0.040  0.044  0.043  0.016  0.999  1.000
       9.21   7.32   517.9    11.0     8.6  2.011  0.063  0.063  0.068  0.025  0.998  0.999
       7.32   6.40   262.0     9.7     8.9  1.560  0.101  0.094  0.108  0.039  0.995  0.999
       6.40   5.81   200.9    10.5    10.0  1.411  0.136  0.124  0.146  0.052  0.992  0.998
       5.81   5.40   209.4    11.7    11.2  1.381  0.143  0.136  0.153  0.055  0.991  0.998
       5.40   5.08   223.0    13.0    12.5  1.325  0.147  0.134  0.158  0.057  0.991  0.998
       5.08   4.82   242.5    14.3    13.7  1.324  0.149  0.138  0.160  0.058  0.991  0.998
       4.82   4.61   230.7    15.3    14.8  1.250  0.163  0.150  0.175  0.063  0.990  0.997
       4.61   4.44   223.3    16.3    15.9  1.165  0.173  0.158  0.186  0.067  0.989  0.997
       4.44   4.28   182.6    17.1    16.8  1.112  0.220  0.203  0.236  0.085  0.982  0.996
       4.28   4.15   137.1    17.7    17.5  0.947  0.282  0.239  0.303  0.110  0.976  0.994
       4.15   4.03   108.8    18.5    18.4  0.882  0.357  0.296  0.383  0.139  0.965  0.991
       4.03   3.92    86.1    19.6    19.5  0.968  0.502  0.421  0.539  0.197  0.944  0.986
       3.92   3.83    72.0    20.5    20.5  0.959  0.637  0.546  0.685  0.251  0.908  0.976
       3.83   3.74    62.9    21.1    21.1  0.755  0.675  0.570  0.726  0.266  0.879  0.967
       3.74   3.66    63.9    22.2    22.1  0.997  0.758  0.732  0.816  0.300  0.813  0.947
       3.66   3.59    47.7    22.3    22.3  0.770  0.934  0.721  1.006  0.371  0.831  0.953
       3.59   3.52    45.5    22.4    22.4  0.711  0.972  0.764  1.047  0.388  0.793  0.941
       3.52   3.46    35.2    22.7    22.7  0.832  1.293  1.075  1.396  0.522  0.677  0.898
       3.46   3.40    30.2    22.3    22.3  0.813  1.434  1.189  1.550  0.585  0.621  0.876
  All reflections    224.9    17.4    16.6  1.189  0.177  0.097  0.190  0.070

  	R linear = SUM ( ABS(I - <I>)) / SUM (I)
     	R square = SUM ( (I - <I>) ** 2) / SUM (I ** 2)
    	Chi**2   = SUM ( (I - <I>) ** 2) / (Error ** 2 * N / (N-1) ) )
     	In all sums single measurements are excluded
```

**Reviewer 2**

1) The authors reasonably point out that despite many manuscripts claiming antibodies block conformational changes associated with fusion, there is little direct evidence this is their mechanism of action. In my view, the best study in this area was published by the laboratory of the late Michael Rossman. Here, they incubated West Nile virus (WNV) with fragments of the monoclonal antibody E16 and then lowered the pH. What they observed was some motion of the E protein bound to Fab, but not the complete low-pH-mediated fusion process. They concluded the antibody trapped the virion in a radial expanded form. A similar approach would be definitive here. Coating ZIKV with Fab and lowering the pH should preserve the antiparallel dimer structure of the E proteins on the virion. This would formally demonstrate “cross-linking” as a mechanism.

Here the authors use mutations in the antigen binding surface of recombinant antibodies to selectively disrupt binding to either of the two E proteins of the antiparallel dimer. With this approach, the authors demonstrate that residues in domain II (site one) are the most critical for binding, as mutation of the paratope in a manner predicted to disrupt these interactions failed to bind and neutralize infection. Mutation of the antibody in a manner predicted to disrupt site two interactions did not appear to impact binding but markedly changed neutralization. Mutations predicted to disrupt both sites reduced binding and neutralization. While this was an interesting approach, the reagents are uncharacterized to confirm the mutations conferred the intended changes in binding and how this in turn impacted other facets of antibody binding with the potential to impact antibody function, including angle of approach. It was confusing that a different set of mutations was used here for site one- what are the impact of D43A and D54A alone on site one? The confidence intervals provided from the curve fitting of a single experiment is not an appropriate measure of uncertainty- independent experiments are required. What is the impact on neutralizaiton if you create mutations on Zika at site two?

*Response: Our studies demonstrated that paratope mutations that ablate interactions with the minor binding site (site 2) on E, have a minimal impact on binding and a major impact on ZIKV neutralization. This observation is consistent with cross dimer binding being critical for the neutralization mechanism. This reviewer suggests additional experiments to directly observe the impact of G9E binding on low pH induced E protein conformational changes on the viral surface leading to membrane fusion. In collaboration with Dr. Sheemei Lok at Duke-NUS, we have completed the studies proposed by the reviewer. We used cryo-EM to test if G9E or its paratope mutants could block pH-triggered fusion and included the in the revised manuscript (see lines 250-277 and figure 6). Briefly, we coated ZIKV with WT G9E Fab or its paratope mutants at pH 8.0 and subsequently lowered the pH from 8.0 to either 6.5 (early endosome pH) or 5 (late endosome pH). As shown in Fig 6, the uncomplexed virus particles appeared spiky and formed aggregates, indicative of E protein fusion peptide exposure and insertion into adjacent viral particles at pH 5. WT Fab G9E bound to the virus particles and prevented aggregation at pH 5, demonstrating that the mechanism of Fab G9E mediated ZIKV neutralization is by cross-linking of the E protein. G9E-Site1 with disrupted EDII lateral ridge interaction in site 1 failed to bind to the virus at any pH and did not block low pH triggered fusion.  G9E-Site1/2 with the mutations targeting  “R252” interaction in site 1  and the entire site 2 interaction bound to ZIKV but failed to block the virus aggregation at pH 5.   In comparison, G9E-Site2 with the mutations abolishing binding to just the minor binding site (Site 2) on the adjacent E protomer, while preserving binding to the main site (Site 1) on EDII bound to ZIKV at pH 8 and 6.5 but failed to block the virus aggregation at pH 5. These observations suggest that the ability of G9E to cross-link E homodimer via site 2 is required for preventing low pH-triggered fusion of ZIKV particles, and site 1 binding alone lacked fusion inhibitory function.*

*We have attempted to generate ZIKVs with targeted mutations in site 1 or 2, but these strains have been difficult to recover suggesting the mutations have a major effect on viral fitness.*

***Minor Issues***

Error bars, stats, and the number of independent experiments requires definition throughout.

*Response: In the revised manuscript, we have defined the error bars and replicates in the figure legends.*

**Reviewer 3**

1) Uncovering the mechanisms by which antibodies neutralize infectious virus is important for the design and development of effective vaccines and antiviral strategies. Especially in the flavivirus field where different classes of antibodies can induce immune-enhancement, provide serotype specific neutralization, or broadly neutralizing cross-reactivity.

The monoclonal antibody G9E was initially identified and described in the Cooper et al 2019 JCI publication. In this previous publication, G9E was cloned from the MBC of a ZIKV infected patient and was shown to potently neutralize ZIKV with specificity to the EDE1 region. In this current manuscript Adams et al further define the binding site of the G9E antibody and demonstrate that glycosylation at site 67 determines ZIKV specificity. The authors also demonstrate that antibody binding across the E-dimer is critical for neutralization capacity.

Given the demonstrated specificity of the G9E antibody to Zika virus and its overlapping footprint with previously described EDE antibodies, this manuscript lacks appeal and novelty to be of broad interest to the viral research community. However this manuscript is very well written and the conclusions are strongly supported by the data.

*Response: This reviewer commented that the paper was well-written and that our conclusions were strongly supported by the data. We disagree with the reviewer that our work lacks novelty because the G9E footprint overlaps with previously described EDE human MAbs. Simply describing the footprint/ epitope of a Mab does not reveal mechanism of neutralization. As noted by both other reviewers, while investigators have previously described human neutralizing antibodies that bind across the flavivirus dimer (EDE, 2D22, ZV117), investigators have not been able to evaluate the roles of overall binding affinity versus E dimer cross linking to the neutralization mechanism. Our study reveals that E protein cross-linking (and not binding affinity) is the main mechanism of neutralization.*

2) Within the introduction, the authors should further highlight the original findings of the G9E antibody. Include the original isolation from the MBC of ZIKV infected patient, its specificity to the EDE epitope, and the neutralization activity across multiple ZIKV viral strains, but not DENV.

*Response: In our previous publication, we observed that high concentrations of EDE1 MAbs had a modest impact (~30% maximum inhibition) on the binding of G9E to Zika virus. While these results suggest some overlap in epitopes, they also highlight the fact that the epitopes are quite distinct. In the revised manuscript, we have expanded the introduction to cover the relationship of these epitopes and the specificity of G9E to ZIKV but not DENV. (see lines 64-68).*

Editors

Although there are numerous criticisms that the reviewers present and that you should respond to, a major point raised is to formally demonstrate the mechanism of neutralization is by crosslinking. This would raise the importance of this study.

*Based on the recommendation of reviewer #2 and the editors, over the past year we developed new reagents and collaborated with Dr. Sheemei Lok to use CRYO-EM to test if G9E binding across the dimer (cross-linking) was the main mechanism of neutralization. These new results support our hypothesis that antibody mediated cross-linking is critical for blocking low pH-triggered fusion of ZIKV particles.*
