# Supplementary material for: Structure and neutralization mechanism of a human antibody targeting a complex Epitope on Zika virus
Source: PLoS Pathog. 2023 Jan 10;19(1):e1010814. doi: 10.1371/journal.ppat.1010814 (PMC9870165; doi:10.1371/journal.ppat.1010814)
Supplement: Attachment — Submitted filename: Response to review for second revision.doc [file ppat.1010814.s011.doc]

**Response to reviewers’ comments [PPATHOGENS-D-21-00433]**

All reviewer comments are in regular text and our response is in italics blue text.

**Reviewer 1**

Figures with neutralization/ binding data still need clarification regarding independent experiments vs technical replicates, etc. Specifically:

1) Figure 3: The legend states that the data in panels B-D represents the average of two measurements. This does not specify whether these were independent experiments or technical replicates in one experiment. Error bars are not addressed. The data in Figure 3D is the basis for the following statement on lines 163-164: “Our results demonstrate that mAb G9E defines an antigenic region centered on EDII that is a major target of serum neutralizing antibodies in ZIKV patients.” While this may be true, it is too strong of a conclusion based on the information provided. Was only a single neutralization assay performed with technical replicates, or two independent experiments? Were the samples tested pairwise? There are no error bars.

*Response: We have revised the figure legend to indicate the number of independent experiments and technical replicates supporting the figure. Moreover, as suggested by the reviewer, we have performed a paired test (Wilcoxon matched pairs test) to compare the ability of human immune sera to neutralize WT and D67N mutant Zika viruses. Nine of 10 subjects had lower titers to the glycan mutant (P = 0.04). Figure 3D has been revised to show the pairwise comparison.*

2) Figure 4B and C: Again, are the measurements from a single experiment or two independent experiments?

*Response: We have revised the figure legend to indicate the number of independent experiments and technical replicates supporting the figure.*

3) Figure 5: The differences in neutralization curves would be easier to see if they were on the same graph, and additionally graphed as EC50 values. Still unclear on what is shown in panels B and C regarding # experiments and error bars. Are the neutralization curves a representative experiment of the three experiments used to calculate the average EC50 values in D? Same question with the binding data.

*Response:* *ZIKV neutralization curves shown as multiple panels in Figure 5B for G9E WT and paratope mutant Fabs are now presented in a single graph as suggested. Panel B and C in Figure 5 are representative nonlinear curve fits of a single experiment with technical replicates for each titration point. Error bars for each data point in panels B and C are standard deviations of the technical replicates. The neutralizing and binding data summary presented in panel D are the means and standard deviations of EC50 values obtained from three (binding) or two (neutralizing) independent experiments with technical replicates for each titration point. EC50 values from the representative curves shown in panels B and C were also used in calculating means and standard deviations in panel D. We have added text to clarify these in Figure 5 legend.*

4) Supplementary Figure 1: What are the EC50 values? This could be clearly depicted by plotting EC50 values on a separate graph.

*Response:* *As suggested, we have included EC50 values in Supplementary Figure 1.*
